# Differentially culturable Mycobacterium tuberculosis in cough-generated aerosols of patients with pulmonary tuberculosis DCTB in cough-generated aerosols

**DOI:** 10.1099/jmm.0.002027

**Published:** 2025-06-06

**Authors:** Luiz Guilherme Schmidt Castellani, Manuela Negrelli Brunetti, Mariana Abou Mourad Ferreira, Taline Canto Tristão, Pedro Sousa de Almeida Júnior, Edward C. Jones-López, Kevin P. Fennelly, Michael R. Barer, Jerrold J. Ellner, Reynaldo Dietze, Moisés Palaci

**Affiliations:** 1 Núcleo de Doenças Infecciosas, Centro de Ciências da Saúde, Universidade Federal do Espírito Santo, Vitória, Brazil; 2 Division of Infectious Diseases, Department of Medicine, Keck School of Medicine of USC, University of Southern California, Los Angeles, CA, USA; 3 Section of Infectious Diseases, Department of Medicine, Boston University School of Medicine and Boston Medical Center, Boston, MA, USA; 4 Pulmonary Branch, Division of Intramural Research, National Heart, Lung, and Blood Institute, National Institutes of Health, Bethesda, MD, USA; 5 Department of Respiratory Sciences, University of Leicester, Leicester, UK; 6 Department of Medicine, Rutgers New Jersey Medical School, Newark, NJ, USA

**Keywords:** cough-generated aerosols, culturability, culture filtrate, differentially culturable, *Mycobacterium tuberculosis*

## Abstract

**Introduction.** While *Mycobacterium tuberculosis* cells in sputum in sputum have been studied extensively, little is known of their properties in exhaled aerosols.

**Hypothesis.** As differentially culturable tubercle bacteria (DCTB) are readily found in sputum, we hypothesized that DCTB might also be present in aerosols and potentially contribute to transmission.

**Aim.** To test cough aerosols from recently diagnosed pulmonary tuberculosis (TB) patients for DCTB.

**Methodology.** Cough-generated aerosols and sputum samples were collected from active pulmonary TB patients (*n*=27). A cough aerosol sampling system was modified to include both an Andersen Cascade Impactor using solid agar and a BioSampler liquid impactor. We performed the most probable number of assays to detect DCTB, using media supplemented with *Mycobacterium tuberculosis* culture filtrate (CF).

**Results.** Briefly, 63% of patients (*n*=17) had advanced TB, and 55.6% (*n*=15) had a 3+ sputum smear for acid-fast bacilli. Evidence for DCTB was found in 8 patients’ aerosols (29.5%) and more than half of the 19 sputum samples tested (*n*=10; 52.6%). Two patients had DCTB in only one of the collected samples (cough aerosols or sputum). Among cough aerosol specimens, two patients (7%) only had CF-dependent DCTB.

**Conclusion.** We detected DCTB in sputum and evidence for their presence in cough samples from pulmonary TB patients. These data suggest that bacilli undetected by traditional mycobacterial cultures may be aerosolized from pulmonary TB patients.

## Introduction

Tuberculosis (TB) transmission results from factors such as the infectiousness of the source case, environmental conditions and both the immune status and the pulmonary ventilation rate in exposed human hosts [1,2]. Risk factors for transmission include the sputum bacillary concentration and cough frequency in the source case and the proximity or duration of the exposure [1]. In a cohort in Uganda, cough aerosol cultures of *Mycobacterium tuberculosis* (MTB) were the strongest predictor of new infections among household contacts [1].

A recent systematic review indicated that infected individuals are highly variable as transmission sources and emphasized the need to define the basis for this variation [3]. The authors noted that between 2 and 31% of individuals were estimated responsible for 80% of transmission at the population level. The dose of pathogens received by susceptible people depends on factors such as the number of bacilli present in aerosol particles of respirable sizes and pathogen survival [4]. While colony-forming bacilli in cough aerosols have been clearly demonstrated [1], the potential that bacilli resistant to recovery by conventional culture may contribute to TB transmission has not been studied.

Both *in vivo* [5] and *in vitro* [6] studies have demonstrated that differentially culturable tubercle bacteria (DCTB) do not form colonies when inoculated onto solid media (also described as non-plateable) but grow in broth cultures supplemented with MTB culture filtrates (CFs) containing resuscitation-promoting factors (RPFs) and other factors that may also contribute [7]. Regular culture and molecular methods do not distinguish between growing metabolically active bacilli and those in altered metabolic states, including DCTB [8]. Consequently, in previous studies [2,9, 10], those bacteria that require CF of liquid culture obtained from actively growing MTB or recombinant RPF for their growth would not have been detected in the aerosol samples. Although some authors have demonstrated high numbers of MTB with these requirements in sputum [6], their occurrence in aerosol has yet to be determined.

## Methods

**Fig. 1.**
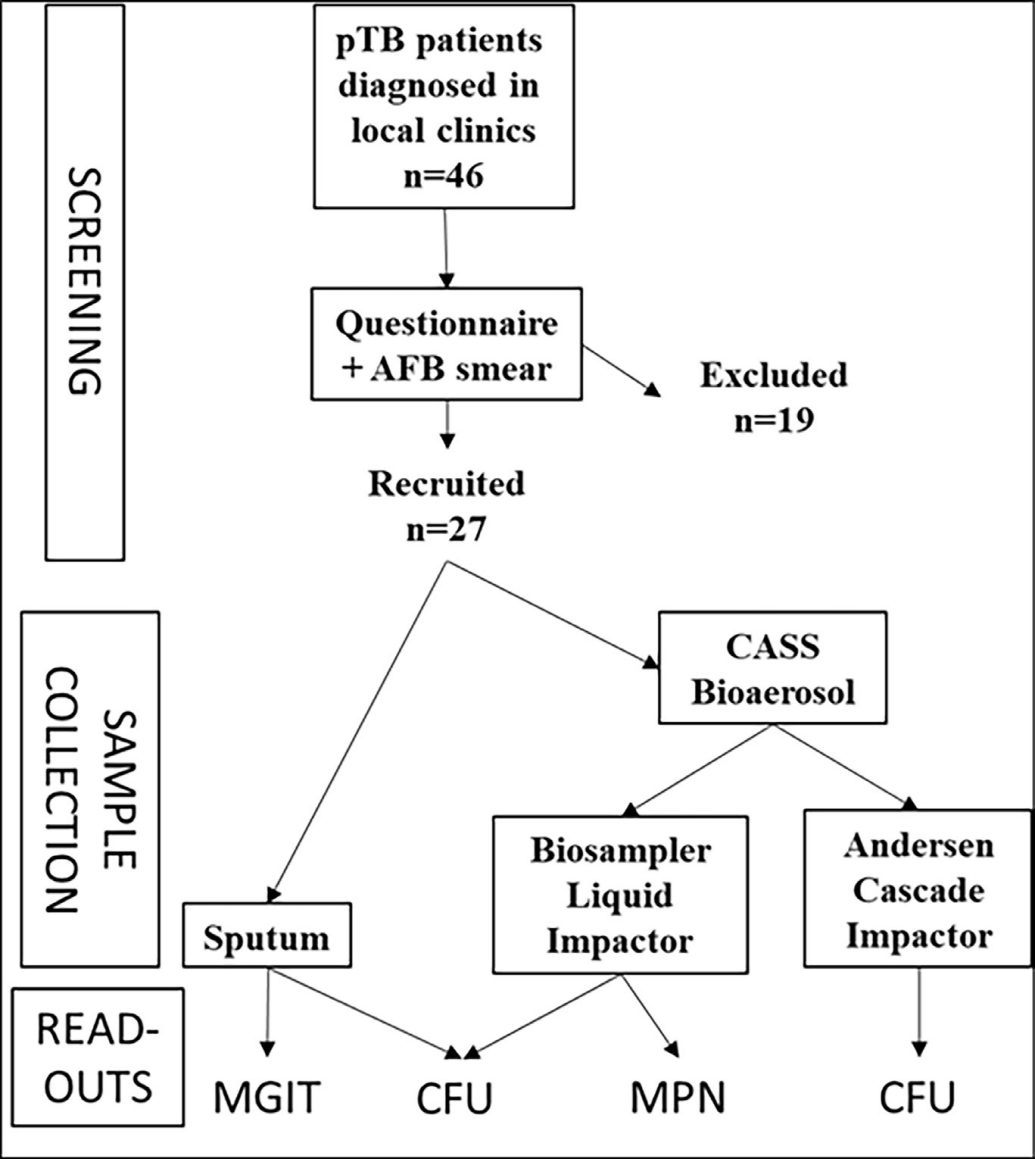
Study design to detect dormant MTB bacilli, activated by CF, from cough aerosol cultures of pulmonary TB patients. Initially, patients were screened through a questionnaire, AFB smear and the molecular test ‘GeneXpert’ result. Then, considering inclusion and exclusion criteria, eligible patients had their sputum collected for (**i**) quantitative culture, (ii) positivity on the Mycobacteria Growth Indicator Tube (MGIT) system and (iii) for the LDM to estimate dormant and active bacilli. Also, the CASS assay was performed, adapted with a bioaerosol sampler for dormant bacilli detection and with the Andersen Cascade Impactor for active bacilli quantification.

This is an observational, cross-sectional study that was designed to detect and quantify the DCTB in cough aerosol cultures of pulmonary TB patients (Fig. 1).

Patients were enrolled at the Núcleo de Doenças Infecciosas of the Universidade Federal do Espírito Santo, located in Vitória, Brazil. They were part of a study from the *International Collaboration in Infectious Diseases Research*, designed to investigate the extent of MTB transmission in household contacts exposed to an index case of infectious pulmonary TB [11]. During the selection of subjects, the following inclusion criteria were considered: a minimum of 18 years old; episodes of coughing for three or more weeks; a new TB episode with, at least, one sputum specimen positive for acid-fast bacilli (AFB) (≥2+) and MTB detected through the GeneXpert test. We excluded patients with a previous history of TB treatment, HIV-infected patients (or those who refused HIV testing), and those who were too ill to consent or unable to understand the study protocol.

Patients provided sputum specimens for AFB smear microscopy with auramine O fluorescent stain, solid culture with Ogawa-Kudoh’s method and liquid culture on BD Bactec^™^ MGIT^™^ 960 System. All positive cultures were tested for AFB by Ziehl–Neelsen stain and for the presence of MTB complex using the rapid immunochromatographic test SD Bioline TB Ag MPT64 (SD, Seoul, South Korea).

Cough aerosol sampling was performed in a negative pressure HEPA filtered room with controlled temperature and humidity. Patients underwent a single aerosol sampling procedure at 7 a.m., after having their cough severity evaluated through a peak flow meter and an analogue scale [12,13]. Patients coughed into the cough aerosol sampling system (CASS) for two sessions of 5 min each, with 5 min of rest in between sessions. The CASS was set up as previously described [14], except that it included only one six-stage Andersen Cascade Impactor [10,14] for plateable bacilli counting on solid agar and a BioSampler^®^ (SKC Ltd., Dorset, UK) for DCTB detection and quantification in liquid media [15]. The Andersen Impactor was loaded with solid agar plates of Middlebrook 7H11 supplemented with 200 UI ml^−1^ polymyxin B, 10 µg ml^−1^ amphotericin B, 50 µg ml^−1^ carbenicillin and 20 µg ml^−1^ trimethoprim. The PANTA^™^ antibiotic mixture (BD BBL^™^, New Jersey, USA) was added at double concentration [16] to 4 ml of 7H9 liquid media in the BioSampler to suppress the growth of contaminating bacteria. After cough aerosol collection, CASS was unloaded aseptically in a biological safety cabinet and then disinfected by autoclave cycle and 70% ethanol for 1 h. Plates were incubated at 37 °C, followed by weekly inspection until week 6, for c.f.u. counts.

CF preparation – First, we performed a pre-culture using the reference strain H37Rv (ATCC^®^ 27294^™^) in Middlebrook 7H9 medium (1:20 v/v) supplemented with 10% (v/v) OADC, 0.2% (v/v) glycerol and 0.05% (v/v) Tween^™^ 80. Next, the pre-culture was incubated at 37 °C under agitation (100 r.p.m.) until reaching the OD of 0.5 at 580 nm. Then, the volume of this pre-culture was transferred to a new culture medium (1:10 v/v) until the OD_580 nm_ reached 0.8. The culture was centrifuged for 15 min at 3,000 ***g*** and the supernatant was sterile filtered (0.22 µm). To ensure the properties of the components present, the CF was lyophilized and stored at −80 °C until use, not exceeding 6 months. For the sterility control of the filtrate, we performed the Ziehl–Neelsen technique and inoculated an aliquot of the final product on blood agar and 7H11 plates.

The limiting dilution method (LDM) was performed, as previously described [6,17], to obtain the most probable number (MPN) of cough aerosol bacillary load. CF contains growth factors to reactivate the metabolism of non-plateable cells, allowing the estimation of the CF-dependent DCTB population [17]. Briefly, in a 48-well microplate, the first four columns were used as a conventional MPN test and contained only Middlebrook 7H9 broth. The other four columns were the supplementation with CF, to assess both plateable c.f.u. and DCTB [8]. We added 0.5 ml of the BioSampler content in each of the eight wells of line A (1:2 dilution). Then, a ten-fold serial dilution was performed and 10 µl aliquots of these dilutions were plated on 7H11 plates for c.f.u. count [6]. For MPN calculation, we used a file freely available online [18]. The presence and proportion of DCTB bacilli were assessed by calculating the resuscitation index (RI) determined by the difference between the log_10_ MPN count per ml in the presence of CF and the log_10_ c.f.u. per ml on 7H11 agar in the same sample [17]. Where c.f.u. counts were negative together with a positive 7H9+CF culture, a value of 10 c.f.u. ml^−1^ was assigned to allow calculation of the RI. Where the resultant RI was <0.3, the sample was designated ‘possible’ positive for DCTB. Allowing for the imprecision inherent in MPN and c.f.u. determinations, we have used an RI of 0.3, representing a twofold excess of 7H9+CF over 10 c.f.u. as the threshold defining ‘probable’ DCTB.

For c.f.u. counting from sputum, the total sample was decontaminated and digested with NaOH and *N*-acetyl-l-cysteine. Next, it was serially diluted and inoculated into selective Middlebrook 7H11 plates, incubated at 37 °C for up to 42 days, and was weekly examined for contamination and colony counting. The c.f.u. counts were also performed on 7H11 from the BioSampler dilutions as indicated above.

All statistical analyses were performed using the IBM SPSS Statistics (IBM Corp.) and the R statistical package (RStudio version 1.2.1335). Statistical tests and significance were referred to in each figure legend.

## Results

Twenty-seven patients with pulmonary TB were eligible to participate in this study (Table 1). The mean age was 36 years; 81.5% were male (*n*=22) and 18.5% were female (*n*=5). Seventeen patients (63%) had a far advanced stage of the disease, assessed by an expert physician through the interpretation of a radiographic exam. Fifteen patients (55.6%) had an AFB smear grade of 3+, nine (33.3%) had a 2+ and three (*n*=11.1%) had a 1+.

**Table 1.** Demographic information on 27 pulmonary TB patients

Characteristic	*N*	%
Mean age in years (min–max)	36 (19–73)	–
Sex		
Male	22	81.5
Female	5	18.5
Body mass index (min–max)	20.8 (16.2–27.45)	–
Radiographic severity of disease:		
Minimal	2	7.4
Moderately advanced	8	29.6
Far advanced	17	63
Sputum AFB smear grade*		
Scanty	0	0
1+	3	11.1
2+	9	33.3
3+	15	55.6

*Sputum smear was graded as scanty (1–9 AFB in 100 fields), 1+ (10–99 AFB in 100 fields), 2+ (1–10 AFB per field in 50 checked fields), 3+ (more than 10 AFB per field in 20 checked fields).

**Fig. 2.**
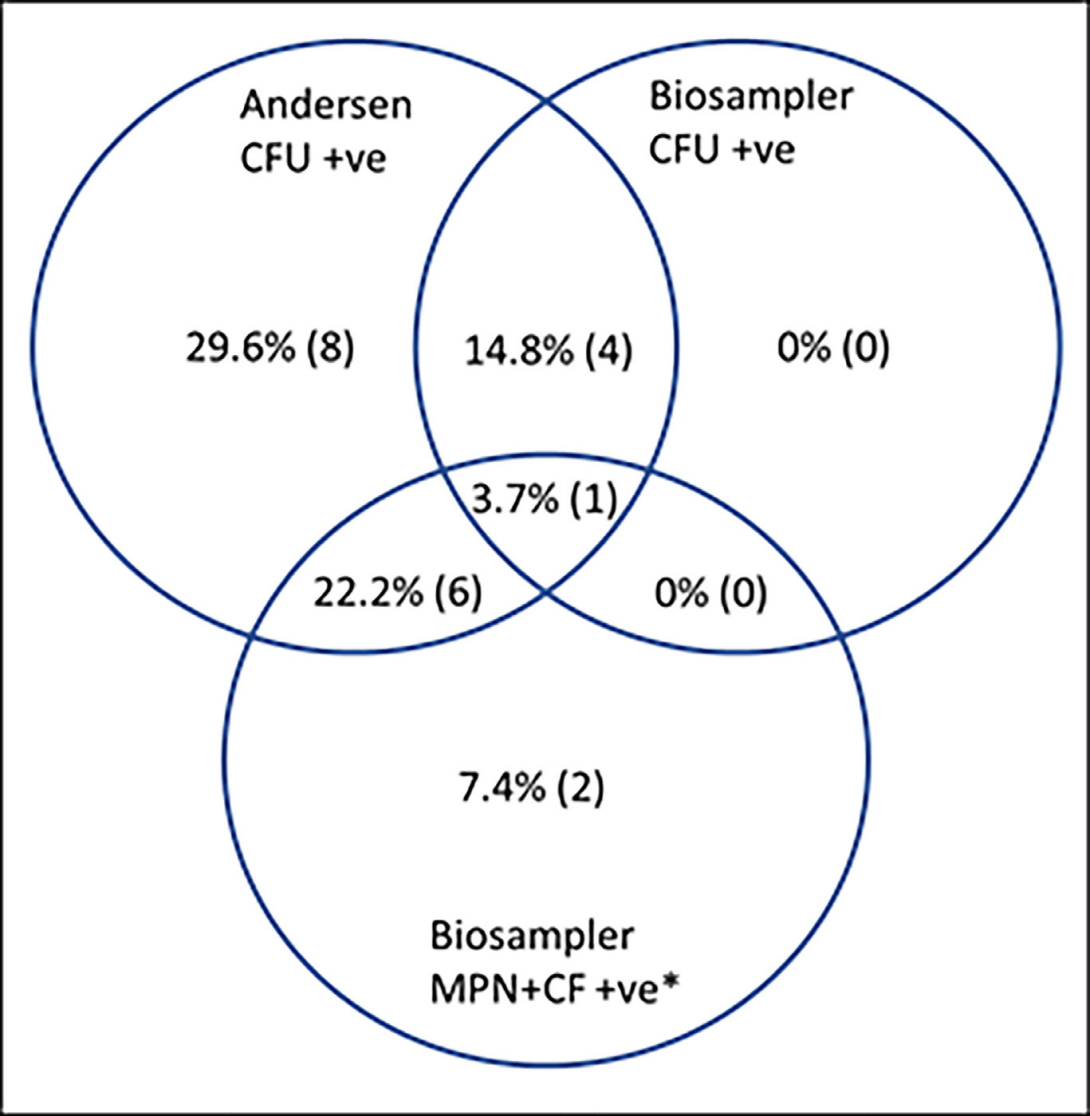
Proportion of MTB bacilli recovered by different assays applied to CASS samples. Twenty-one patients produced at least one positive culture in their aerosol samples. Six patients produced no positive aerosol cultures. *Biosampler MPN+CF counts for one patient were excluded for technical reasons. Percentages are shown against the 27 participants, with numbers of individuals in parentheses.

Table 2 shows the results obtained from cough aerosols produced by 27 patients and sputum samples from all but 7. For the aerosol assessments, 19 (70.4 %) were positive in the Andersen Cascade, 5 (18.5 %) in the Biosampler c.f.u. count and 9 (33.3 %) in the Biosampler MPN count with CF supplementation (one MPN count was unavailable). The correspondence in positivity between these assays is shown in Fig. 2.

**Table 2.** Culturable MTB output in aerosols generated by cough from pulmonary TB patients.

	CASS samples					Sputum samples				
	Andersen^*^	Biosampler				MGIT	Quantitative culture (Log_10_ values)			
PID	7H11^#^	7H11	7H9+CF^†^	RI^‡^		TTP (h)^§^	7H11	7H9	7H9+CF^†^	RI
*Andersen c.f.u. positive – DCTB negative, RI<0 (n=1*)										
11	49	200	3	<0		nt	nt	nt	nt	nt
*Andersen c.f.u. positive, 7H11 c.f.u. negative, DCTB possible, RI<0.30 (n=5*)										
19	85	0	2	<0		93	5.5	≥6.1	6.1	0.6
1	4	0	1	<0		122	4.6	4.6	5.8	**1.2**
2	333	0	4	<0		NT^¶^	nt	nt	nt	nt
10	201	0	4	<0		80	5	4.9	5.4	0.4
8	3	0	12	0.08		nt	nt	nt	nt	nt
***7H11 c.f.u. negative, DCTB probable, RI>0.30 (n=3)***										
24	33	0	24	0.38		148	3.4	0.6	3.2	−0.2
27	0	0	24	0.38		nt	nt	nt	nt	nt
25	0	0	560	1.75		236	nt	nt	nt	nt
*Andersen or 7H11 c.f.u. positive, DCTB negative (n=12*)										
22	106	0	0	<0		118	5.4	0	4.9	−0.5
23	17	0	0	<0		114	6.2	0	1.4	−4.8
3	57	0	0	<0		nt	5.8	2.4	5.4	−0.4
4	203	200	0	<0		105	4.2	5.1	5.6	**1.4**
5	98	100	0	<0		114	5.8	1.9	5.8	0
6	67	0	0	<0		131	5	1.4	5.2	0.2
16	3	0	0	<0		113	nt	nt	nt	nt
26	1	0	0	<0		128	4.7	4.7	4.9	0.2
12	41	100	0	<0		149	5.1	1.5	1.7	−3.4
13	75	200	0	<0		110	4.7	1.5	3	−1.7
All aerosol cultures negative										
7	1	0	0	<0		256	4.7	4.9	4.7	0
14	8	0	0	<0		nt	nt	nt	nt	nt
17	0	0	0	<0		140	0	0	6.2	**5.2**
20	0	0	0	<0		103	5.1	5.2	5.7	0.6
21	0	0	0	<0		172	4	1.1	1.1	−2.9
18	0	0	0	<0		158	4.5	1.3	4.7	0.2
9	0	0	0	<0		141	4.3	nt	nt	nt
15	0	0	0	<0		106	3	CC^¶^	CC^¶^	na

*Andersen Cascade.

†Middlebrook 7H9 supplemented with CF.

‡RI.

§Time-to-positivity.

¶Culture contamination.

#All 7H11 cultures denote c.f.u. counts.

Andersen samples reflect the total c.f.u. count from the cascade, Biosampler and quantitative culture values are per ml of sample. RI values (Log_10_7H9+CF – Log_10_7H11 c.f.u.) identify whether samples contain DCTB bacilli. RI values above 0 and <0.9 have been considered borderline and those >0.9 positive.

na, not applicable; nt, not tested; PID, patient identifier.

Biosampler samples that were negative for c.f.u. but positive by MPN+CF potentially demonstrate the presence of DCTB. By the criteria stated above, we classify five (18.5 %) samples as possibly DCTB positive and three (11%) as probably so. A relatively high limit of detection for the c.f.u. count precludes definitive recognition of DCTB in these samples. Nonetheless, evidence consistent with their presence was found in 29.5% of the participants in this study. Comparing DCTB results where CASS and sputum were available (18 patients, Table 2), there was no significant association between the two results, i.e. sputum positivity did not predict CASS positivity (*P*>0.99, Fisher's exact).

In 6 of the 27 patients (22.2%), no bacilli were recovered by any assay on aerosol collection. However, growth was observed in the sputum of these six patients; in one case (PID17), only DCTB was detected.

There was no correlation between time-to-positivity and evidence for DCTB (*P*>0.05).

## Discussion

To our knowledge, this is the first evidence that cough-generated aerosols from pulmonary TB patients contain MTB cells dependent on CF for their growth. Evidence for DCTB in cough aerosols was found in nearly one-third of our participants (29.5%).

We applied stringent criteria to classify samples as possibly or probably DCTB positive, or negative, based on the excess of culturable bacteria in the limiting dilution count over colony-forming cells in the same sample. It is, however, possible that all samples contained some DCTB, but their presence was obscured by an excess of colony-forming bacilli. Heterogeneity in bacillary phenotypes within a population is a well-known strategy increasing the likelihood of survival against multiple stresses [19].

The metabolic states of DCTB bacilli are not well defined, and their relationships to recognized states such as hypoxia-induced dormancy and persisters have yet to be defined [8]. Moreover, their relationship to multiple non-replicating MTB states recognized as dormancy in a recent review is uncertain [20]. Nonetheless, it can generally be agreed that DCTB are non-replicating and show a reduction in metabolic activity that is reversible in at least a proportion of the cells present. While some refer to DCTB as dormant, the bacilli so termed are clearly distinct from the well-characterized bacterial endospore. However, they do show several related features, including stress and antimicrobial tolerance [21]. Non-replicating and resistant forms such as spores, plant seeds and protozoan cysts are a common feature in the dissemination stage of many life forms.

Pulmonary TB is an infectious disease transmitted by aerosols produced, often associated with cough [1]. Our assays detected mycobacterial growth in the cough of 77.8% of patients. Additionally, our results showed the presence of DCTB in 52% of the patients studied, detected through cough aerosols, sputum or both.

The presence of culturable bacilli in cough has been shown previously [4], including after starting treatment [22]. Studies that evaluated only the active bacilli obtained 28% positivity in the cough aerosol of the studied patients [14]. By providing the possibility of growth of differentially culturable subpopulations, our study achieved 77% positivity from coughing. This illustrates the value of including assessment of DCTB in cough samples. While the addition of CF generally increased counts in sputum samples, the supplemented MPN gave a lower number than 7H11 c.f.u. counts from the same sample in six instances, as indicated by negative RI values. This apparent inhibitory activity of CF for some samples has been noted in previous studies but remains to be defined [6].

The relevance of these low metabolic DCTB on TB pathogenesis remains unknown, including the actual ability to infect. Some studies have shown that bacilli submitted to low concentrations of oxygen were able to invade human alveolar tissue. This shows that the presence of dormant bacilli in cough aerosol can be a form of transmission [23]. Another study showed that MTB with negative AFB was associated with higher infectivity, and smear-negative bacteria in untreated patient specimens may be a non-staining, slow-metabolizing phenotype better adapted to airborne transmission [24].

In two patients, bacilli were only detected by MPN+CF in the cough aerosol, with no c.f.u. forming bacilli being detected, and therefore, they would not have been diagnosed by the conventional method. The presence of dormant bacilli was also observed in the cough of another patient who did not present this population in the sputum. In studies that evaluated the presence of MTB populations that were not recovered in culture, they observed that this population increases after the start of treatment [25]. Furthermore, the high percentage of recovery bacilli, observed in our aerosol and sputum, represents a concern for the control of TB, reinforcing the value of detecting this population.

The patients in this study have different ages, with a predominance of males. Studies have shown that men are more predisposed to TB than women around the world, and this can be explained by biological factors, greater exposure to risk factors (e.g. men smoke more than women) and health care [26]. Regarding the radiographic severity of disease, most patients were diagnosed as far advanced (63%). No correlation was found between severity and the presence or absence of DCTB, both in cough aerosol and in sputum. We note also that the presence of DCTB in sputum did not associate with their presence in CASS. Higher numbers of participants in a fully powered study maximizing sensitivity for DCTB detection would be required to address this point.

Although more difficult to perform, the diagnosis based on cough could be an option for patients who cannot produce sputum. In this study, five patients did not produce sputum, and in all of them, it was possible to detect growth from coughing, with evidence for DCTB in three of them. For conventional testing, these patients would have to undergo a sputum induction. This procedure, though not invasive, can cause discomfort and is contraindicated in people with notes, patients with aneurysms and those with compromised lung function or thoracic trauma [27]. Other less resource-demanding and cough-independent samples, such as tongue swabs or facemask samples, could provide alternatives in these cases [28,29].

Some authors have discussed possible relationships between DCTB and bacteria termed by some to be in a ‘viable but non-culturable (VBNC)’ state. As discussed over 25 years ago in this and other journals [30,31], we do not favour the use of this term in an operational (as opposed to a conceptual) domain. Since they are cultured, albeit by a special method, DCTB were never non-culturable. This is also true of ‘VBNC’ studies where recovery has been demonstrated by any culture technique, rendering the term oxymoronic [30]. Where recovery in culture is not demonstrated, viability, as defined as the capacity for further propagation [31], must be considered doubtful.

Our study has some limitations that must be considered. First, some tests were not performed on many patients because of their incapacity to produce sputum. Also, this study was performed with no repetition, which can amplify random errors. In fact, due to our limited sample number, our data are restricted to untreated patients, but it has been well demonstrated that survival mechanisms that lead to non-replicating states may be induced by antimicrobial exposure, among other factors [32]. Therefore, as future perspectives, new studies should consider the quantification of DCTB during antibiotic treatment and among cases of TB recurrence and treatment failure. Additional studies should also be done to know the real infectivity and specific antimicrobial sensitivity profile of this dormant population so it can be targeted in the future.

In conclusion, we present evidence for the presence of dormant CF-dependent MTB in cultures from pulmonary TB patients’ coughs. In addition, we show that some patients had bacilli growth exclusively in cultures supplemented with CF. Clinically, this represents the necessity to assess the metabolically different bacilli with appropriate culture methods. Also, the mechanisms underlying MTB metabolic shifts remain unclear and require further investigation.

## Abbreviations

AFB: acid-fast bacilli
CASS: cough aerosol sampling system
CF: culture filtrate
DCTB: differentially culturable tubercle bacteria
LDM: limiting dilution method
MGIT: Mycobacteria Growth Indicator Tube
MPN: most probable number
MTB: *Mycobacterium tuberculosis*
RI: resuscitation index
RPF: resuscitation-promoting factor
TB: tuberculosis
VBNC: viable but non-culturable
